# Screening and Identification of Key Microenvironment-Related Genes in Non-functioning Pituitary Adenoma

**DOI:** 10.3389/fgene.2021.627117

**Published:** 2021-04-27

**Authors:** Jing Guo, Qiuyue Fang, Yulou Liu, Weiyan Xie, Chuzhong Li, Yazhuo Zhang

**Affiliations:** ^1^Beijing Neurosurgical Institute, Capital Medical University, Beijing, China; ^2^Department of Neurosurgery, Beijing Tiantan Hospital Affiliated to Capital Medical University, Beijing, China; ^3^Beijing Institute for Brain Disorders Brain Tumor Center, Beijing, China; ^4^China National Clinical Research Center for Neurological Diseases, Beijing, China

**Keywords:** non-functioning pituitary adenoma, tumor microenvironment, immune, invasive, recurrence

## Abstract

**Purpose:**

Non-functioning pituitary adenoma (NFPA) is a very common type of intracranial tumor, which can be locally invasive and can have a high recurrence rate. The tumor microenvironment (TME) shows a high correlation with tumor pathogenesis and prognosis. The current study aimed to identify microenvironment-related genes in NFPAs and assess their prognostic value.

**Methods:**

73 NFPA tumor samples were collected from Beijing Tiantan Hospital and transcriptional expression profiles were obtained through microarray analysis. The immune and stromal scores of each sample were calculated through the ESTIMATE algorithm, and the patients were divided into high and low immune/stromal score groups. Intersection differentially expressed genes (DEGs) were then obtained to construct a protein–protein interaction (PPI) network. Potential functions and pathways of intersection DEGs were then analyzed through Gene Ontology and the Kyoto Encyclopedia of Genes and Genomes. The prognostic value of these genes was evaluated. The quantitative real-time polymerase chain reaction in another set of NFPA samples was used to confirm the credibility of the bioinformatics analysis.

**Results:**

The immune/stromal scores were significantly correlated with cavernous sinus (CS) invasion. The Kaplan–Meier curve indicated that the high immune score group was significantly related to poor recurrence-free survival. We identified 497 intersection DEGs based on the high vs. low immune/stromal score groups. Function enrichment analyses of 497 DEGs and hub genes from the PPI network showed that these genes are mainly involved in the immune/inflammatory response, T cell activation, and the phosphatidylinositol 3 kinase-protein kinase B signaling pathway. Among the intersection DEGs, 88 genes were further verified as significantly expressed between the CS invasive group and the non-invasive group, and five genes were highly associated with NFPA prognosis.

**Conclusion:**

We screened out a series of critical genes associated with the TME in NFPAs. These genes may play a fundamental role in the development and prognosis of NFPA and may yield new therapeutic targets.

## Introduction

Non-functioning pituitary adenomas (NFPAs) are benign pituitary neoplasms. They account for 14–54% of pituitary adenomas, which are the second most common primary intracranial tumor (Fontana and Gaillard, 2009; Fernandez et al., 2010; Ostrom et al., 2015; Day et al., 2016; Ntali and Wass, 2018). The delay in the diagnosis of NFPAs is related to the clinical manifestations of the lack of excessive hormone secretion. Apart from pituitary incidentalomas, NFPAs are usually detected when the tumor is large enough to cause pressure effects on surrounding structures, with symptoms including headache, visual field defects, decreased libido and hydrocephalus (Lindholm et al., 2006; Sam et al., 2015). Surgery is preferred when treatment is needed and can be combined with adjuvant radiotherapy (Wass and Karavitaki, 2009). Although NFPAs are benign neoplasms, most demonstrate invasive growth at the time of diagnosis, manifesting as local infiltration, and have an increased risk of post-operative recurrence (Landeiro et al., 2015). However, the current understanding of the molecular mechanism that causes NFPAs and the mechanism of their invasion is still limited. Therefore, exploring the pathogenesis of NFPAs is essential for early detection of the disease and early prevention of invasive NFPAs.

Recently, increasing numbers of studies have shown that the tumor microenvironment (TME) plays a vital role in tumor progression and treatment (Tahmasebi Birgani and Carloni, 2017; Wu and Dai, 2017; Ren et al., 2018; Bian et al., 2019). The TME is the environment in which a tumor exists, including its blood vessels, lymphatic vessels, extracellular matrix, stromal cells, immune/inflammatory cells, and secreted proteins (Balkwill et al., 2012). Among the components of the TME, immune cells (lymphocytes and macrophages) and stromal cells (such as fibroblasts) are the main non-tumor cells and they could have an impact on cancer progression and may represent a new therapeutic target (Liu et al., 2018; Xiong et al., 2018; Ocana et al., 2019). For example, the poor prognosis of renal cell carcinoma is related to the infiltration of baseline CD8 T-cells (Giraldo et al., 2015; Drake and Stein, 2018). Some studies have found that macrophages can promote the invasiveness of pituitary tumors (Marques et al., 2019; Sato et al., 2019; Yagnik et al., 2019), as well as prostate (Maolake et al., 2017), ovarian (Colvin, 2014) and breast cancer (Carron et al., 2017). Kashima et al. (2019) have shown that the accumulation of tumor-associated fibroblasts (TAFs) in esophageal squamous cell carcinoma can enhance lymph node metastasis and promote tumor progression. Moreover, Lv et al. (2018) found that the expression of α-smooth muscle actin and vascular endothelial growth factor in invasive pituitary adenoma TAFs was higher than that in non-invasive TAFs and normal fibroblasts, and TAFs in invasive pituitary adenoma promoted the proliferation of GH3 cells *in vitro* and tumor growth *in vivo*. The above studies suggest that a comprehensive understanding of the TME and its associated genes may be important in improving therapeutic strategies for NFPAs. Based on the specific gene expression signatures of immune and stromal cells, Yoshihara et al. (2013) proposed a new algorithm called Estimation of Stromal and Immune cells in Malignant Tumor tissues using Expression data (ESTIMATE), which infers the proportion of stromal and immune cells in tumor samples by calculating immune and stromal scores. Some researchers have used this method to identify diagnostic and prognostic markers and immune related therapeutic targets in their studies (Jia et al., 2018; Luo et al., 2019; Pan et al., 2019).

In the current study, we used the ESTIMATE algorithm to calculate the stromal score and immune score in the TME of NFPAs to determine prognosis and target genes for NFPA treatment. Based on a high and low immune/stromal score, we obtained a series of differentially expressed genes (DEGs), and their relationship with the invasiveness and prognosis of NFPAs was investigated.

## Materials and Methods

### Patients and Samples

We collected 73 specimens from patients diagnosed with NFPA who underwent surgery and did not receive pre-operative radiochemotherapy at Beijing Tiantan Hospital from October 2007 to July 2014. These patients lacked clinical and biochemical evidence of adenohypophysis hormone overproduction. Post-operative recurrence of NFPA was defined as an increase of more than 2 mm in the diameter of the largest tumor measured through magnetic resonance imaging (MRI) in any direction from the day of surgery to the end of follow-up. Based on immunohistochemistry results, the NFPAs were classified into gonadotroph adenomas (GAs), silent adenomas (SAs), and null cell adenomas (NCAs). Cavernous sinus (CS) invasion was defined using the Knosp grading scale (grade 3 or 4) based on pre-operative MRI results (Knosp et al., 1993). Based on MRI data, the NFPAs were classified into microadenomas (diameter <10 mm), macroadenomas (≥10 mm) and giant adenomas (≥40 mm). The clinical data for the adenomas are described in Table 1. In addition, 15 CS invasive and 11 non-invasive NFPA clinical samples from the same hospital were collected as an independent validation cohort to verify the expressive levels using quantitative real-time polymerase chain reaction (qRT-PCR) (Supplementary Table 1). Tumor specimens were stored in liquid nitrogen within 30 min after resection until RNA was extracted. This study was approved by the local Ethics Committee and informed consent was obtained from each subject.

**TABLE 1:** Summary of patient demographics and clinical characteristics.

Characteristic	Number of patients
**Gender**	
Female	39
Male	34
**Age, years**	
≤52	41
>52	32
**Histological types**	
GAs	41
SAs	29
NCAs	3
**CS Invasion**	
Yes	34
No	39
**Tumor size classification**	
Macroadenoma	53
Giant adenoma	20
**Recurrence**	
Yes	27
No	46

*GAs, gonadotroph adenomas; SAs, silent adenomas; NCAs, null cell adenomas; CS, cavernous sinus.*

### Total RNA Extraction and RNA Microarrays

A phenol-free mirVana^TM^ miRNA isolation kit (Cat # AM1561, Ambion, Austin, TX, United States) was used to extract and purify total RNA for the generation of fluorescently labeled SBC human ceRNA array V1.0 cRNA targets (4 × 180 K). The labeled cRNA target was then hybridized with the slide. After hybridization, slides were scanned using an Agilent microarray scanner (Agilent Technologies, Santa Clara, CA, United States). After extracting the data using feature extraction software 10.7 (Agilent Technologies, Santa Clara, CA, United States), a quantile algorithm was used to normalize the raw data using the Limma software package for the R program.

### Differentially Expressed Genes (DEGs) Based on Immune/Stromal Scores

We applied the ESTIMATE algorithm in the R program (version 3.5.3) to calculate the immune and stromal scores for each sample (Yoshihara et al., 2013). To test the correlations between prognoses and immune/stromal score, NFPAs were classified into a high-immune/stromal score group and a low-stromal/immune score group. The R package “maxstat” was used to identify the optimal cutoff for grouping patients. CIBERSORT (Newman et al., 2015) is a deconvolution algorithm^1^ used to calculate the abundance of 22 types of human infiltrated immune cells in each sample. DEGs were identified by using the R program to compare the high-immune/stromal score group and the low-stromal/immune score group, and further study was considered with a | fold change| >2 and a *p*-value < 0.05. Venn diagrams were used to depict the common DEGs in both groups.

### Construction of a Protein–Protein Interaction (PPI) Network

The protein–protein Interaction (PPI) network of DEGs was constructed using an online STRING database^2^ (Version 11.0) (Szklarczyk et al., 2019), and the interaction with a combined score >0.9 was considered statistically significant. The visualization and analysis of PPIs were completed using Cytoscape (version 3.3.0) software (Shannon et al., 2003). The degree distribution was analyzed using the Cytoscape plug-in and Network Analyzer.

### Functional Enrichment Analysis

All of the intersection DEGs were enrichment analyzed through Gene Ontology (GO) and the Kyoto Encyclopedia of Genes and Genomes (KEGG) to predict their biological function, which was performed using the ClueGo (Bindea et al., 2009) Cytoscape plugin (version 3.3.0). GO terms analysis included biological process, cellular components (CCs), and molecular functions (MFs) terms and a KEGG pathway with an adjusted *p*-value < 0.05 was considered to be significant.

### Evaluation of Intersection DEGs

Box-plots were used to depict the intersection gene expression levels between the CS invasive and non-invasive groups. The statistical significance of the difference between the groups was calculated by using a *t*-test. Kaplan–Meier plots and log-rank test *p*-values were used to clarify the correlation between each intersection gene and prognostic performance. A forest plot was used to visualize the independent risk factors of the intersection genes for recurrence-free survival (RFS) through Cox regression analysis.

### Validation of Gene Expression Through qRT-PCR

The total RNA of the validated samples was extracted and purified as described above. Then, 1 μg of total RNA was reverse transcribed into complementary DNA (cDNA) using a High Capacity cDNA Reverse Transcription Kit (0049472, Thermo Fisher) in accordance with the manufacturer’s instructions. Power SYBR^TM^ Green PCR Master Mix (4367659, Thermo Fisher) was used for qRT-PCR with a total reaction volume of 20 μL. The housekeeping gene glyceraldehyde-3-phosphate dehydrogenase (*GAPDH*) was used as an internal control gene. All primers were synthesized by Sangon Biotech (Shanghai, China). The sequences of the primers are shown in Table 2. The level of mRNA was determined using QuantStudio 3 and 5 Systems (Applied Biosystems). For relative quantitation, expression levels were calculated using the 2^–ΔΔ*CT*^ method. All qRT-PCR analyses were performed in triplicate.

**TABLE 2:** Primers used for qRT-PCR.

Gene	Forward primer (5′-3′)	Reverse primer (3′-5′)
*ABCA8*	AAAACAGACCGCGTGATCCT	CGCACTTTAGCTTCCCTTGG
*GPC3*	GCCGAATGCTCACCAGAATG	CACATTGCAGTAACCGCCAC
*TFPI2*	ACGATGCTTGCTGGAGGATAG	TCTGTGGACCCCTCACACTG
*MFAP4*	GCTCCCACCTCTCTTATGCC	CGGATTTTCATCTCAGTGCGTTT
*GAPDH*	GCCATCACTGCCACTCAGAAGA	ATGACCTTGCCCACAGCCTTG

## Results

### The Correlation of Immune/Stromal Scores With Clinical Characteristics in NFPA

**FIGURE 1:**
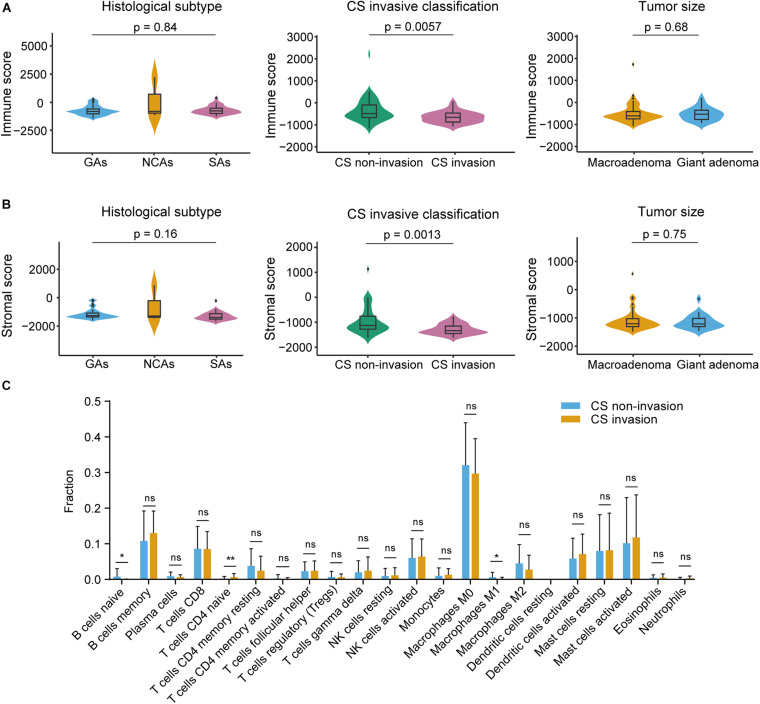
Immune and stromal scores were not correlated with histological subtype, tumor size, but were correlated with cavernous sinus (CS) invasion. **(A)** Distribution of immune scores plotted against histological subtype (*p* = 0.84), CS invasive classification (*p* = 0.0057), tumor size (*p* = 0.68). **(B)** Distribution of stromal scores plotted against histological subtype (*p* = 0.16), CS invasive classification (*p* = 0.0013), and tumor size (*p* = 0.75). **(C)** CIBERSORT analysis of the proportions of 22 tumor infiltrating immune cell types between CS invasion and CS-non-invasion group (1000 permutations). *p < 0.05; **p < 0.01; ns, not significant.

The RNA-seq data consisted of 73 samples, including 34 male and 39 female subjects with the median age at diagnosis was 52 year (range 25–73 year). The median follow-up was 60 months (range 4–98 months). We obtained complete gene expression profiles through high throughput sequencing and identified 18827 mRNAs expressed in NFPA with an expression value > 0 in the 73 samples. The stromal scores ranged from −1648.8 to 1273.7, the immune scores ranged from −1429.1 to 2376.3, and the ESTIMATE scores ranged from −2866 to 3650, as calculated using the ESTIMATE algorithm. The distribution of the immune and stromal scores did not vary by histological subtype (*p* = 0.84/0.16) and tumor size (*p* = 0.68/0.75) but was significantly different from that of the CS invasive and CS non-invasive groups (*p* = 0.0057/0.0013) (Figures 1A,B). The degrees of immune cell infiltration of each sample were determined by the CIBERSORT algorithm (Supplementary Figure 1). We found that the infiltration degree of B cells naive, T cells CD4 naive, and Macrophages M1 were significantly different between CS invasion and CS non-invasion group (Figure 1C). The data were analyzed using the Kruskal–Wallis test and the Mann Whitney test.

**FIGURE 2:**
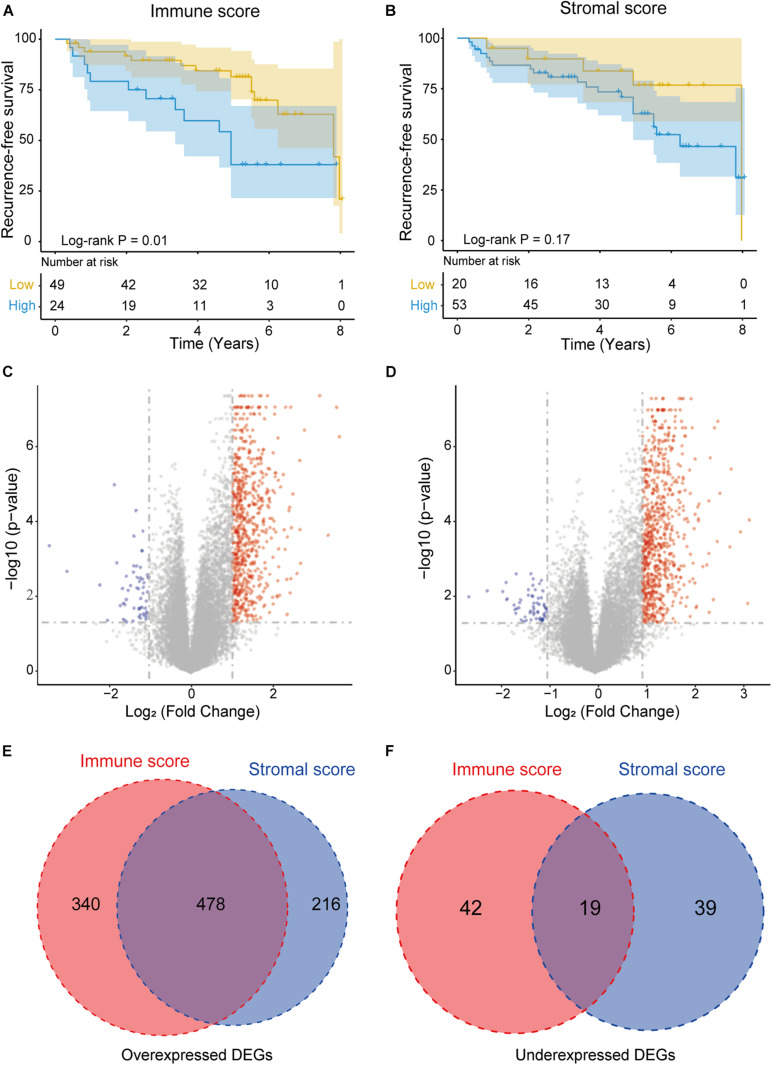
Immune conditions are associated with NFPA recurrence-free survival (RFS) and identification of DEGs based on immune scores and stromal scores. **(A)** Low immune scores were significantly correlated with an improved RFS (*p* = 0.01). **(B)** Stromal scores were not significantly associated with RFS (*p* = 0.17). **(C,D)** Volcano plot of DEGs from the low vs. high immune score/stromal score groups. Genes with *p* < 0.05 are shown in red (fold change >2) and blue (fold change <–2). Black plots represent the remaining genes (those with no significant difference). **(E,F)** Venn diagrams showing the number of commonly upregulated or downregulated DEGs in the stromal and immune score groups.

To investigate the relationship between prognosis and the immune/stromal score, 73 NFPAs were classified into high-score groups and low-score groups (see methods) and analyzed through Kaplan–Meier survival analysis and the log-rank test. The results revealed that the low immune scores were significantly correlated with better RFS (*p* = 0.01, Figure 2A). The low stromal scores were consistently correlated with better RFS, although this correlation was not statistically significant (*p* = 0.17, Figure 2B).

### DEGs for the Immune/Stromal Scores in NFPA

By comparing the gene expression profiles of the high and low immune score groups, we identified 879 immune-related DEGs (cut-off criteria: *p* < 0.05 and | fold change| >2), including 818 upregulated genes and 61 downregulated genes (Figure 2C). Similarly, based on the high- and low-stromal score group, we identified 694 upregulated and 58 downregulated stromal-related genes (Figure 2D). In addition, Venn diagram analysis revealed that there were 478 intersection upregulated genes and 19 intersection downregulated genes in both the immune and stromal groups (Figures 2E,F). These intersection DEGs were selected for subsequent analysis.

### Functional Enrichment Analysis of Intersection DEGs

**FIGURE 3:**
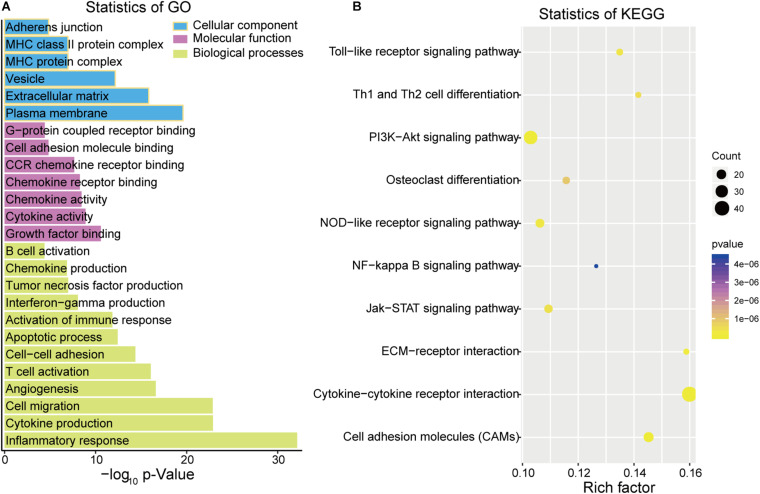
Function analysis of DEGs by Gene Ontology (GO) **(A)** and Kyoto Encyclopedia of Genes and Genomes (KEGG) **(B)**.

Based on the ClueGO gene annotation tool, we conducted GO analysis to further evaluate the biological functions of the 497 intersection DEGs (Figure 3). Three subontologies of these DEGs were analyzed (Figure 3A). In terms of biological processes (BP), these intersection DEGs were mainly enriched in terms of T cell activation, inflammatory response, cell migration, and cytokine production; for the CC, these DEGs were primarily clustered in the extracellular matrix, major histocompatibility complex (MHC) protein complex and adherens junction; with regard to MF, they were mainly associated with cytokine activity, cell adhesion molecule (CAM) binding, and chemokine receptor binding. Moreover, enrichment analysis of the KEGG pathway suggested that they are mainly related to the phosphatidylinositol 3 kinase-protein kinase B (PI3K-Akt) signaling pathway, CAMs, and cytokine-cytokine receptor interaction (Figure 3B).

### PPI Network Construction of Intersection DEGs

**FIGURE 4:**
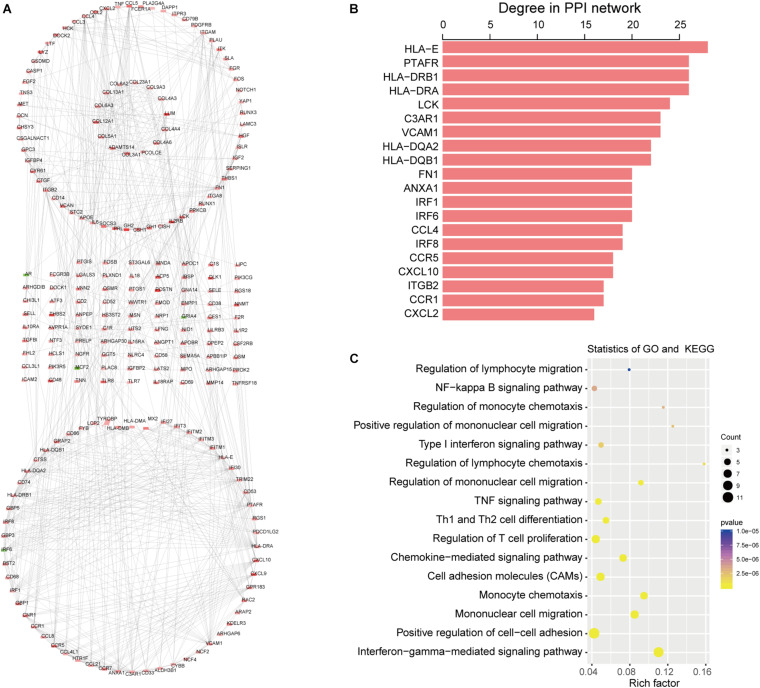
Topological features of DEGs PPI network. **(A)** The view of the PPI network. Red indicates upregulation and green indicates downregulation. **(B)** The key DEGs in the PPI network with the top 20 degree distributions. **(C)** Function analysis of the 20 DEGs in the PPI network.

The PPI network was constructed based on 497 intersection genes to explore hub genes and develop a thorough picture of these genes at the systems level. It contains 220 nodes and 154 interactions (Figure 4A). Figure 4B shows the top 20 DEGs based on degree distribution in the PPI network. The topological analysis suggested that *HLA-E* (degree = 28) was the key gene in the PPI network, which could interact with most genes as it had the highest degree. Moreover, functional enrichment analysis of these top 20 DEGs by ClueGo analysis also showed that they were significantly associated with 63 GO terms and 22 KEGG pathways, such as regulation of lymphocyte migration, regulation of T cell migration, NF-kappa B signaling pathway and TNF signaling pathway (Figure 4C).

### The Expression of Stromal-Immune Score Related Intersection DEGs in NFPA

**FIGURE 5:**
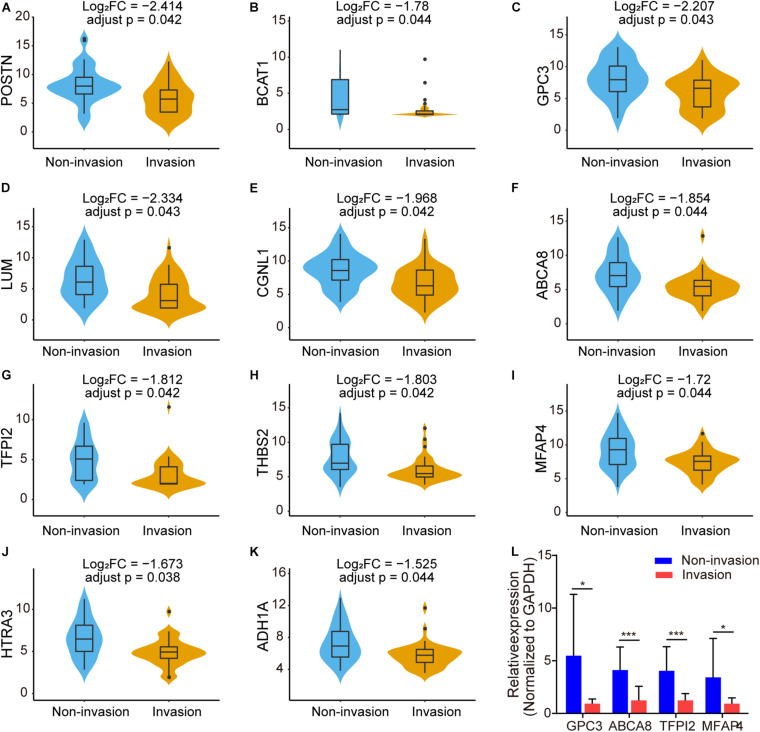
Box plots showing the different expression level of 11 genes with adjust *p* < 0.05 and | Log_2_(fold change)| >1.5 in NFPA CS invasion status [**(A–K)**, FC: fold change]. **(L)** Validation of differentially expressed genes in the NFPAs through qRT-PCR. Relative expression of GPC3, ABCA8, TFPI2, and MFAP4 between CS invasive NFPAs and non-invasive NFPAs. Data were analyzed using unpaired Student’s *t*-test. *p < 0.05; ***p < 0.001.

Among the 497 intersection genes (478 upregulated genes and 19 downregulated genes), there are 88 genes which are significantly differentially expressed in the CS invasive group and non-invasive group (*p* < 0.05, | Log_2_(fold change)| >1). Among these genes, there are 11 genes (*POSTN*, *LUM*, *GPC3*, *CGNL1*, *ABCA8*, *TFPI2*, *THBS2*, *BCAT1*, *MFAP4*, *HTRA3*, and *ADH1A*) with adjust *p* < 0.05 and | Log_2_(fold change)| >1.5 (Figures 5A–K).

Because of the limited sample size, *GPC3*, *ABCA8*, *TFPI2*, and *MFAP4* were randomly selected from the above genes to verify their expression levels in our clinical samples. We performed qRT-PCR to measure the expression level of these genes between CS invasive NFPAs and non-invasive NFPAs (Figure 5L). Consistent with the results of the microarray data analysis, *GPC3*, *ABCA8*, *TFPI2*, and *MFAP4* were significantly downregulated in the invasive group (*n* = 15) compared with the non-invasive group (*n* = 11).

### Survival Analysis of Intersection DEGs

**FIGURE 6:**
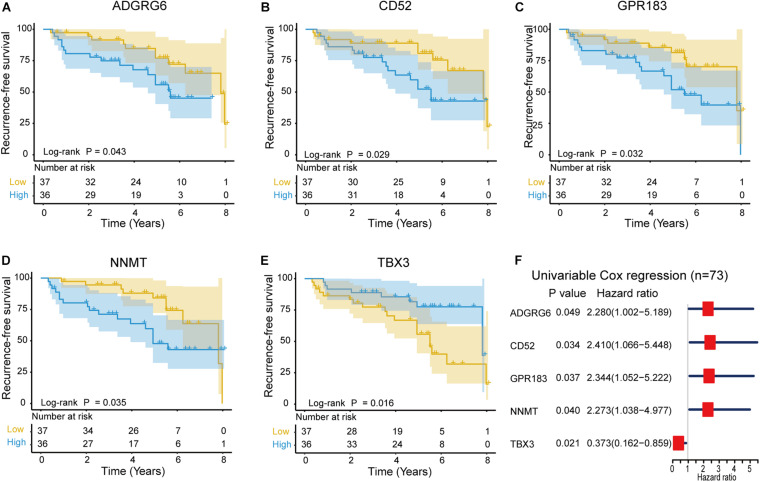
Kaplan–Meier survival curves showing the impact of the expression level of five genes in terms of NFPA recurrence-free survival (RFS). **(A–E)** Comparison of RFS in the high (red line) and low (blue line) gene expression groups. *p* < 0.05 was used to assess differences in the Log rank test. **(F)** Forest plot of hazard ratios for these five prognostic DEGs. Hazard ratios and corresponding 95% confidence intervals were assessed through univariable Cox analysis.

The correlation between the intersection DEGs and RFS was evaluated through Kaplan–Meier survival curves using the 73 NFPAs. Among the 497 intersection DEGs, five were predictive of RFS (*p* < 0.05), as assessed through the log-rank test (Figures 6A–E). Among these five prognostic intersection DEGs, patients with upregulated *ADGRG6*, *CD52*, *GPR183*, and *NNMT* expression demonstrated shorter RFS time than those with downregulated expression, while upregulated *TBX3* was associated with favorable outcomes. Moreover, a forest plot demonstrated the prognostic effects of these five DEGs through univariable Cox regression analysis (Figure 6F).

## Discussion

Despite advances in endoscopic techniques, most patients with NFPA are at increased risk of post-operative recurrence and a poor therapeutic response because of its invasive growth in surrounding normal tissues at the time of diagnosis (Landeiro et al., 2015; Fernandez-Miranda et al., 2018). Therefore, it is important to explore the pathogenesis and invasiveness of NFPAs, to help us understand their recurrence and provide systematic treatments. The TME is implicated in the initiation and development of various tumors (Balkwill et al., 2012; Hui and Chen, 2015), including pituitary adenomas (Lu et al., 2015; Lv et al., 2018; Sato et al., 2019; Yagnik et al., 2019). Therefore, it is important to identify TME-related genes that are involved in NFPA recurrence and invasiveness.

First, the immune score and stromal score were obtained using the ESTIMATE algorithm. Our results indicate that the immune/stromal score was not significantly correlated with histological subtype and tumor size, but was significantly related to CS invasion. We also investigate the abundance of immune cells in NFPA using the CIBERSORT algorithm, which showed that B cells naive, T cells CD4 naive, and Macrophages M1 were significantly different between the CS invasion and CS non-invasion group. Previous studies have shown that TME-related factors are associated with invasive pituitary adenomas, such as the number of M2-macrophages, the number of inflammatory cells, and increased expression of PD-L1 (Qiu et al., 2013; Sato et al., 2019). In addition, Kaplan–Meier survival analysis showed that a lower immune/stromal score was associated with better RFS. Although the stromal score was not significant in this context, possibly because of the small sample size, it also suggested that the TME may have a deep impact on tumor progression. This is consistent with previous studies showing that tumor-associated macrophages, T regulatory cells and mast cells are associated with poor prognosis in many types of cancer (Bingle et al., 2002; Balkwill et al., 2012; Hui and Chen, 2015; Fridman et al., 2017; Kemeny et al., 2020).

We analyzed 497 intersection DEGs (478 upregulated and 19 downregulated) which were obtained from the high vs. low immune/stromal score groups, as the intersection genes are the most highly conserved. Among the 497 intersection genes analyzed using GO terms, we found that most of them were enrichened in the immune/inflammatory response (BP), cytokine activity (MF) and extracellular matrix (CC). KEGG pathway enrichment analysis showed that these intersection DEGs were mainly clustered in the PI3K-Akt signaling pathway, CAMs, and cytokine-cytokine receptor interaction. The PI3K-Akt signaling pathway is a signal transduction cascade involved in cell growth and metabolism (Leslie et al., 2001). Long et al. (2019) revealed that COL6A6 could block the PI3K-Akt-pathway to suppress the growth and metastasis of pituitary adenoma. Moreover, we were able to construct a PPI module based on these 497 intersection genes. These hub genes are related to the regulation of lymphocyte migration, the regulation of T cell migration, the NF-kappa B signaling pathway and the TNF signaling pathway, which indicated that these genes are related to immune/inflammation and stromal response. We, therefore, assessed them through function enrichment analysis.

We analyzed 497 interaction genes and identified 88 genes that were significantly differentially expressed between the invasion group and the non-invasion group. To further narrow the scope, we showed 11 genes with adjust *p* < 0.05 and |Log_2_(fold change)| >1.5. As the sample size is limited, *GPC3*, *ABCA8*, *TFPI2*, and *MFAP4* were randomly selected to verify the expression level in CS invasive NFPAs and non-invasive NFPAs through qRT-PCR to confirm the microarray analysis data. We found that the qRT-PCR results matched well with the bioinformation analysis of NFPAs. Several identified genes in our results have previously been reported to play important roles in several types of cancer. Wang et al. (2019) found that the expression of the ABC transporter gene (*ABCA8*) was significantly downregulated in prostate cancer compared with non-cancerous prostate tissues (Demidenko et al., 2015). This is consistent with our qRT-PCR and microarray results showing that the expression level of *ABCA8* was significantly decreased in the CS invasive group vs. the non-invasive group. Guo et al. (2020) found that the increased expression of *ABCA8* is correlated with poor prognosis and is associated with immune infiltration in stomach adenocarcinoma. Glypican-3 (GPC3) is a cell surface proteoglycan anchored by glycosyl-phosphatidylinositol (Pilia et al., 1996). Some studies have developed various immune-related treatment strategies against GPC3 (Sayem et al., 2016), particularly in hepatocellular carcinoma (Sawada et al., 2016; Wu et al., 2017). Tissue factor pathway inhibitor 2 (TFPI2) is a proteolytic enzyme inhibitor with a Kunitz domain that is down-regulated in several types of cancer (Konduri et al., 2001; Rollin et al., 2005; Sato et al., 2005). Gaud et al. (2011) found that TFPI2 produced by non-small lung cancer cells may contribute to the synthesis of matrix metallopeptidase by fibroblasts in the microenvironment. Microfibril associated protein 4 (MFAP4) is frequently downregulated in most human cancers and its high mRNA levels are significantly associated with better outcomes in the early stage in several cancers (Yang et al., 2019). In addition, Niu et al. (2011) have shown that MFAP4 promotes inflammatory cell recruitment and assists immunological cancer surveillance to limit cancer cell survival in early stage cancers.

Finally, five TME-related genes were identified with a significant relationship to the RFS. Among them, the four upregulated genes *ADGRG6*, *CD52*, *GPR183* and *NNMT* correlate with unfavorable NFPA outcomes. ADGRG6 is a member of the adhesion G protein-coupled receptor family and the depletion of *ADGRG6* expression in urothelial bladder carcinoma cells compromised their ability to recruit endothelial cells and induce tube formation (Wu et al., 2019). Upregulated CD52 is correlated with poor survival in patients with lung adenocarcinoma (Li et al., 2018). CD52 is a glycosylphosphatidylinositol-anchored protein expressed on the surface of normal T and B lymphocytes and is also known as the CAMPATH-1 antigen (Hale et al., 1996). In addition, nicotinamide N-methyltransferase (NNMT) was found to be overexpressed in gastric carcinoma tissue compared with adjacent tissues and is related to poor prognosis (Chen et al., 2016). NNMT is a phase II metabolizing enzyme, and mainly catalyzes the methylation of nicotinamide and other pyridines into pyridinium ions (Xie et al., 2014). Moreover, Wang et al. (2019) found that overexpressed NNMT is correlated with poor survival and chemotherapy response in breast cancer patients who received chemotherapy.

As explained above, a series of genes that we identified through bioinformatics methods are related to TME, and these genes are also related to patient prognosis and treatment. Although the function and role of some of these genes have been reported in multiple types of tumor and several genes have been validated in another set of NFPAs, further verification in large and diverse samples is still needed. Moreover, the mechanisms and potential functions of these TME-related genes in NFPAs remain to be investigated.

In summary, TME-related genes might be correlated with tumor invasion and prognosis. Our study may provide a novel signature and therapeutic target for the immune/stromal component in NFPAs.

## Data Availability Statement

The data presented in the study are deposited in the Gene Expression Omnibus (https://www.ncbi.nlm.nih.gov/geo/) with accession numbers: GSE169498, further inquiries can be directed to the corresponding author/s.

## Ethics Statement

The studies involving human participants were reviewed and approved by Ethics Committees of Beijing Tiantan Hospital. The patients/participants provided their written informed consent to participate in this study.

## Author Contributions

WX, CL, and YZ worked on conception, designed the research, and approved the manuscript. QF and YL contributed to collecting and analyzing the clinical data of patients. JG was dedicated to data analysis, interpretation, and drafting. All authors read and approved the final manuscript.

## Conflict of Interest

The authors declare that the research was conducted in the absence of any commercial or financial relationships that could be construed as a potential conflict of interest.

## Footnotes

1 https://cibersortx.stanford.edu/

2 http://string-db.org

## Supplementary Material

Supplementary Figure 1 Stacked bar chart of the fraction of immune cells of the tumor microenvironment in 73 NFPA patients.

Supplementary Table 1 Summary of validated patients demographics and characteristics.
