# Supplementary figures and images for: Screening and Identification of Key Microenvironment-Related Genes in Non-functioning Pituitary Adenoma
Source: Front Genet. 2021 Apr 27;12:627117. doi: 10.3389/fgene.2021.627117 (PMC8110910; doi:10.3389/fgene.2021.627117)

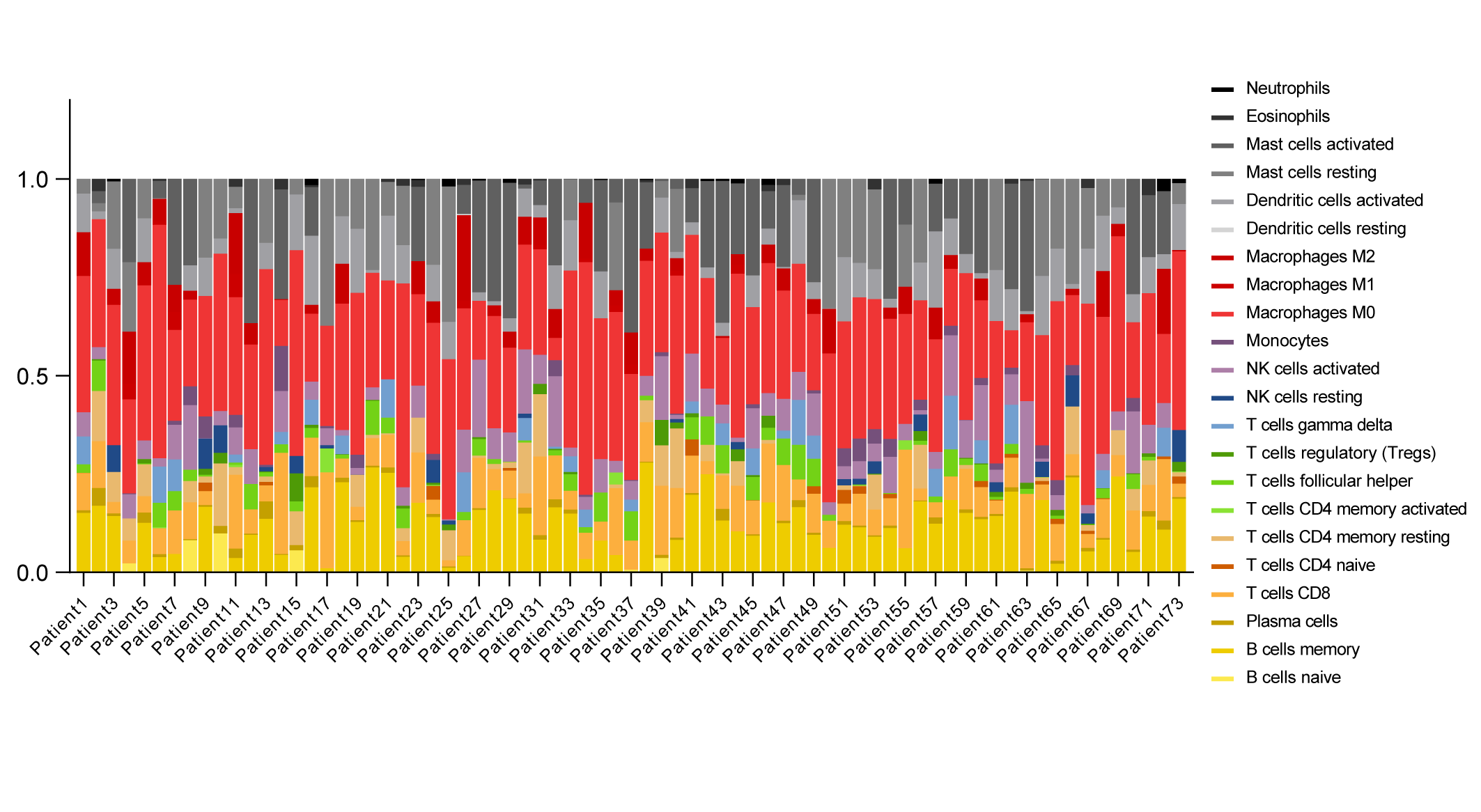

Supplement: Supplementary Figure 1 — Stacked bar chart of the fraction of immune cells of the tumor microenvironment in 73 NFPA patients. [file Image_1.TIF]
