# Supplementary material for: Screening and Identification of Key Microenvironment-Related Genes in Non-functioning Pituitary Adenoma
Source: Front Genet. 2021 Apr 27;12:627117. doi: 10.3389/fgene.2021.627117 (PMC8110910; doi:10.3389/fgene.2021.627117)
Supplement: Supplementary Table 1 — Summary of validated patients demographics and characteristics. [file Table_1.docx]

| **Supplementary table 1: Summary of validated patients demographics and characteristics** | | | |
| --- | --- | --- | --- |
| **Characteristic** | | **Group** | |
|  |  | CS invasive  (n) | CS non-invasive (n) |
| **Sex** |  |  |  |
|  | female | 10 | 3 |
|  | male | 5 | 8 |
| **Age** |  |  |  |
|  | ≤ 52 | 10 | 5 |
|  | > 52 | 5 | 6 |
| **Pathological type** |  |  |  |
|  | GAs | 6 | 7 |
|  | SAs | 8 | 3 |
|  | NCAs | 1 | 1 |
| **Volume classification** |  |  |  |
|  | macroadenoma | 9 | 11 |
|  | gaint adenoma | 6 | 0 |

GAs, gonadotroph adenomas; SAs, silent adenomas; NCAs, null cell adenomas; CS, cavernous sinus.
